# Supplementary material for: Estimated prevalence and gender disparity of physical activity among 64,127 in-school adolescents (aged 12–17 years): A multi-country analysis of Global School-based Health Surveys from 23 African countries
Source: PLOS Glob Public Health. 2022 Oct 21;2(10):e0001016. doi: 10.1371/journal.pgph.0001016 (PMC10021872; doi:10.1371/journal.pgph.0001016)
Supplement: S1 Table — (DOCX) [file pgph.0001016.s001.docx]

**S1 Table:** Leave one sensitivity analysis of the survey years

| Survey country omitted | Survey year | Pooled burden of adolescents’ physical activity level (%) in Africa | 95% CI |
| --- | --- | --- | --- |
| Algeria | 2011 | 20.0 | 17.0-22.0 |
| Benin | 2016 | 19.0 | 17.0-21.0 |
| Botswana | 2005 | 20.0 | 18.0-22.0 |
| Djibouti | 2007 | 20.0 | 18.0-22.0 |
| Egypt | 2012 | 20.0 | 18.0-22.0 |
| Ghana | 2012 | 20.0 | 17.0-22.0 |
| Kenya | 2003 | 20.0 | 18.0-22.0 |
| Liberia | 2017 | 20.0 | 18.0-22.0 |
| Libya | 2007 | 20.0 | 18.0-22.0 |
| Mauritania | 2010 | 20.0 | 18.0-22.0 |
| Mauritius | 2017 | 19.0 | 17.0-21.0 |
| Morocco | 2016 | 20.0 | 18.0-22.0 |
| Mozambique | 2015 | 20.0 | 18.0-22.0 |
| Namibia | 2013 | 20.0 | 17.0-22.0 |
| Senegal | 2005 | 20.0 | 18.0-22.0 |
| Seychelles | 2015 | 20.0 | 17.0-22.0 |
| Sierra Leone | 2017 | 20.0 | 17.0-22.0 |
| Sudan | 2012 | 20.0 | 18.0-22.0 |
| Tanzania | 2014 | 20.0 | 18.0-22.0 |
| Tunisia | 2008 | 20.0 | 17.0-22.0 |
| Uganda | 2003 | 20.0 | 18.0-22.0 |
| Zambia | 2004 | 20.0 | 18.0-22.0 |
| Zimbabwe | 2003 | 20.0 | 18.0-22.0 |
