# Supplementary material for: Estimated prevalence and gender disparity of physical activity among 64,127 in-school adolescents (aged 12–17 years): A multi-country analysis of Global School-based Health Surveys from 23 African countries
Source: PLOS Glob Public Health. 2022 Oct 21;2(10):e0001016. doi: 10.1371/journal.pgph.0001016 (PMC10021872; doi:10.1371/journal.pgph.0001016)
Supplement: S2 Table — (DOCX) [file pgph.0001016.s002.docx]

**S2 Table:** Meta-regression analysis to explore the sources of heterogeneity

| Variable | Meta-regression | |
| --- | --- | --- |
|  | Coefficient | p-value |
| Human Development Index | 0.017 | 0.325 |
| Economic Health | 0.001 | 0.973 |
| Sub-region | 0.007 | 0.452 |
| Survey Year | 0.005 | 0.022* |
| Sample size | -001 | 0.170 |
