# Supplementary material for: Estimated prevalence and gender disparity of physical activity among 64,127 in-school adolescents (aged 12–17 years): A multi-country analysis of Global School-based Health Surveys from 23 African countries
Source: PLOS Glob Public Health. 2022 Oct 21;2(10):e0001016. doi: 10.1371/journal.pgph.0001016 (PMC10021872; doi:10.1371/journal.pgph.0001016)
Supplement: Attachment — Submitted filename: response to reviewers.docx [file pgph.0001016.s003.docx]

**Responses to Reviewers Comments**

I humbly extend my warmest greetings to you and your reputable journal for your swift responses and feedback. I write on behalf of my colleague authors to submit our responses to the invaluable comments provided by your astute experienced reviewer. The format employed follows a point-to-point mode of reply to all comments. Please find below our responses:

Reviewer #1: This is an important analysis of multi country data on physical activity among adolescents in Africa.

Kindly find below my comments:

**Comment:** This important analysis will miss a great opportunity to conduct subgroup analysis on the various sub-regions in Africa and looking at the trend of physical activity over the years.

**Authors’ response:** Thanks for the comment. The authors agree with the reviewer’s recommendation. As a result, further sub-group analyses have been conducted based on Human Development Index (low vs medium vs high vs very high), economic health (low-income countries vs. lower middle-income countries vs. upper middle-income countries vs high income countries), sub-region (North Africa vs West Africa vs Southern Africa vs East Africa), and survey year (2003-2010 vs 2011-2017). This can be found in Figure 4.

**Comment:** What was the age range of the adolescents? Was it similar across countries?

**Authors’ response:** Thanks for the comment. As stated in the manuscript already, the age ranged from 12-17 years and was similar across each country and survey.

**Comment:** Comment on the results of the heterogeneity test

**Authors’ response:** Thanks for the comment. The authors have now commented on the results of the heterogeneity test. It reads ‘’To explore the sources of heterogeneity that existed in estimated physical activity prevalence, meta-regression analysis was performed. The findings showed significant increased trend in the GSHS survey years (coefficient=0.005, p=0.022). However, insignificant trends were found for HDI, economic health, sub-region, and the sample size [p>0.05] (S2 Table)’’……. ‘’The authors have explored the possible source of heterogeneity through sub-group and meta-regression analyses and have discussed accordingly in the results and the discussion sections. The paragraph reads ‘’ Heterogeneity between the surveys’ countries in the pooled prevalence of sufficient physical activity level was high, extending I^2^ >87%. As a result, sources of heterogeneity were further explored through sub-group and meta-regression analyses with respect to HDI, countries’ economic health, sub-region, and survey years. The only possible source heterogeneity was the survey years. For instance, in the sub-group analysis, surveys performed between 2003-2010 had sufficient physical actively level of 18% as compared to survey conducted between 2011-2017 (i.e., 22%). Similarly, the multivariable meta-regression showed increased trends across the survey years’’.

**Comment:** What are the possible sources of heterogeneity from the various countries? Discuss them and how they influence your results.

**Authors’ response:** Heterogeneity between the surveys’ countries in the pooled prevalence of sufficient physical activity level was high, extending I^2^ >87%. As a result, sources of heterogeneity were further explored through sub-group and meta-regression analyses with respect to HDI, countries’ economic health, sub-region, and survey years. The only possible source heterogeneity was the survey years. For instance, in the sub-group analysis, surveys performed between 2003-2010 had sufficient physical actively level of 18% as compared to survey conducted between 2011-2017 (i.e., 22%). Similarly, the multivariable meta-regression showed increased trends across the survey years.

**Comment:** Did you conduct any sensitivity analysis on the pooled prevalence? E.g. leave-one-out analysis? Such results will be very informative.

**Authors’ response:** Thanks for the comment. Initially, sensitivity analysis was not performed however, based on the above recommendation, leave one out sensitivity analysis has now been performed. It reads’’ Leave one out sensitivity analysis was conducted to assess the robustness of the estimates obtained from the meta-analysis’’…… **‘’**Additionally, the authors performed sensitivity analyses by removing one survey at a time and recalculating the pooled effect estimate of the remaining surveys on the pooled burden of sufficient physical activity. The results showed that there is no influential survey on the overall pooled estimate. For instance, the pooled estimated sufficient physical activity level among adolescents in African ranged from 19.0% [95% CI: 17.0%-21.0%] in Benin and Mauritius to 20.0% [95% CI: 17.0%-22.0%]**’’.**

**Comment:** Some of the data was conducted as far back as 2003. How relevant is such information to the pooled prevalence you estimated?

**Authors’ response:** Thanks for the comment. To ensure representativeness, the authors selected every GSHS that have been conducted on the continent. Also, such method has been employed in Guthold et al., 2019 study published in Lancet where surveys as far back as 2001 where included. ‘’Additionally, the authors performed sensitivity analyses by removing one survey at a time and recalculating the pooled effect estimate of the remaining surveys on the pooled burden of sufficient physical activity. The results showed that there is no influential survey on the overall pooled estimate. For instance, the pooled estimates from the sensitivity analyses showed that sufficient physical activity level among adolescents in African ranged from 19.0% [95% CI: 17.0%-21.0%] in Benin and Mauritius to 20.0% [95% CI: 17.0%-22.0%] (S1 Table).’’

**Comment:** Discuss on the impact of the differences in year of the data collection on the pooled prevalence
**Authors’ response:** Thanks for the comment. The impact of the differences in year of the data collection on the pooled prevalence has been reported accordingly. It reads ‘’ the authors performed sensitivity analyses by removing one survey at a time and recalculating the pooled effect estimate of the remaining surveys on the pooled burden of sufficient physical activity. The results showed that there is no influential survey on the overall pooled estimate. For instance, the pooled estimated sufficient physical activity level among adolescents in African ranged from 19.0% [95% CI: 17.0%-21.0%] in Benin and Mauritius to 20.0% [95% CI: 17.0%-22.0%] (S1 Table).’’….’’Furthermore, for GSHS survey year, the pooled estimates for survey conducted from 2003-2010 and 2011-2017 were; 18.0% [95% CI: 16.0%-20.0%], and 21.0% [95% CI: 17.0%-24,0%] respectively’’.

Minor comments
**Comment:** Kindly check the use of abbreviations. Define them on first mention

**Authors’ response:** Thanks for the comment. The use of abbreviation checked and have been defined on first mention.

Reviewer #2: Introduction
**comment 1:** Second sentence of Introduction, if words limit allow, could you give an example of the enormous benefits on health?

**Authors’ response:** Thanks for the comment. As recommended, the authors have now given some examples of enormous benefits of physical activity on health. The sentence now reads ‘’ Despite enormous benefits on health (i.e., improve cardiovascular fitness, muscle strength, bone health, and reduce depression), cognitive development, and prosocial behavior in people aged 11-17 years, the majority of adolescents are considered to be physically inactive globally’’.

**Comment 2:** GSHS abbreviations in the manuscript are not quite consistent, I think you meant “Global School-based student Health Survey”?

**Authors’ response**: The authors would like to thank the reviewer for this observation. The Global School-based student Health Survey (GSHS) abbreviation inconsistency has now been rectified throughout the manuscript.

**Comment 3:** Paragraph 3, last sentence, could you elaborate more on why the lack of representative African adolescents’ PA data is a problem?

**Authors’ response**: Thanks for the comment. The sentence has been elaborated and shifted to the last sentence in paragraph 2. It now reads ‘’ The lack of representative data on adolescents’ (12 to 17 years) physical activity level in Africa are likely to impede effective public health policy, accurate interventions and decision making, as well as blurry the WHO’s target for 15% reduction in physical activity by 2030^1,2^ on the continent. Hence, the current study was motivated and designed to provide the overall baseline data and further explore the gender disparity on sufficient physical activity level among in-school adolescents in Africa’’

Methods
**comment 4:** I suggest the methods section should follow the STROBE guideline. If it is already following the guideline, please state.

**Authors’ response**: The current format is already following the STROBE guidelines hence a sentence has been stated as recommended.

**Comment 5:** I suggest you mention in the Measure subsection that the outcome variable, physical activity, was self-reported. You included this in the strength and limitation but not in the methods.
**Authors’ response**: Thanks for the comment. The self-reported outcome measure has now been mentioned in the method section. it reads ‘’ Physical activity was assessed by a single question and was self-reported’’

Results
**comment 6:** Table 1, could you elaborate on the unit of physical activity level measurement? I am not sure if these are supposed to be minutes, or are they comparable with this format? Also add unit to response rate. Same comment for Table 2.

**Authors’ response**: Thanks for the comment. As stated in the method section, an adolescent was considered as ‘physically active’ if he/she engages in physical activity for at least 60 minutes per day for at least five days in a week. As a result, the outcome was dichotomized as; “sufficient physical activity’’ [i.e., met the recommended ≥5 days per week of physical activity] and “insufficient physical activity’’ [i.e., did not meet the recommended ≥5 days per week of physical activity]. Hence the physical activity reported in Table 1 was the number of adolescents who met the recommended WHO guidelines for physical activity i.e., frequency. Similar assertion holds for Table 2 but Table 2 was reported in percentages with corresponding CI. The unit for the response rate was percentage, and rectified now.

**Comment 7**: Table 1: to make this more intuitive for the reader could the authors arrange with some logic eg alphabetical or in ascending order for any of the columns. Same comment for Table 2.

**Authors’ response**: Thanks for the comment. The Table 1 and 2 have now been arranged in alphabetical order (i.e., Country)

**comment 8:** Table 1, for sample size of each country, I would suggest that you also include the number of populations the sample size represented. Since in the statistical analysis section you mentioned: “The weights allow the results to be extrapolated to the entire study population as well as the entire in-school adolescents’ population in Africa”. So, the reader can get an idea of represented population.

**Authors’ response**: Thanks for the comment. This is a secondary data analysis from World Health Organization, and the original data did not include the overall adolescents’ population in each country. However, adding weights make it possible to generalized the outcome to the overall sampled population from each country i.e., in-school adolescents

**Comment 9:** Figures 1-3, I really like these figures however the image quality is poor. I wonder if this is due to technical issue.

**Authors’ response**: Thanks for the comment. We followed the journal figure quality format using the PACE; therefore, we believe it would be sorted out at their end.

**comment 10:** Figures 2-3, from the methods section, I was not aware that there will be 3 meta-analysis figures. It would be helpful to elaborate how “burden of PA” was generated in both male and females in the methods section.

**Authors’ response**: The authors agree with the reviewer. The above recommendation has been incorporated into the method section. It reads ‘’ Additionally, gender-specific estimates was generated for each country in Africa separately through random effect meta-analysis. Both the overall and gender-specific estimates of sufficient physical activity were represented on forest plots’’

**comment 11:** Figures 2-3, it would be helpful to elaborate what you mean by “burden of PA” in the methods section.

**Authors’ response**: Thanks for the comment. Burden is a general term for prevalence in burden of disease studies or epidemiological studies. It is now explained as legend in Figure 2-3.

**comment 12:** Figures 2-3, would it be possible to overlay these male and female figures so that it would be easier to compare them?

**Authors’ response**: Thanks for the comment. Overlaying is possible however; it will further reduce the image quality hence the separate figures.

**Comment 13:** Given the evidence that inactivity increases with age, and variably by sex, could the authors comment on the age distribution of their sample? And whether there was any different in the age distribution in boys vs girls?

**Authors’ response**: Thanks for the comment. As stated in the manuscript already, the age ranged from 12-17 years and was similar across each country, gender and survey.

Discussion

**comment 14:** I think the second sentence of the previous paragraph and the first sentence of this paragraph says the same thing?

**Authors’ response**: Thanks for the comment and observation. The second sentence of the first paragraph has been revised. It reads ‘’ The study was designed to provide the overall baseline data and further explore the gender disparity on sufficient physical activity level among in-school adolescents in Africa’’

**Comment 15:** I find this sentence “Physical inactivity among adolescents has become a public health epidemic around the world”, rather odd here. I think it would be nice for this sentence “Overall, only 20% of adolescents in Africa met the current WHO physical activity recommendation” to be followed directly by “This conclusion.”

**Authors’ response**: Thanks for the comment. “Physical inactivity among adolescents has become a public health epidemic around the world’’ is now removed and rectified in accordance with the reviewer’s comment.

**Comment 16**: 2nd paragraph, I wonder if you can make this paragraph more concise.

**Authors’ response.** The authors would like to thank the reviewer for the comment. as recommended the paragraph is revised and concise now. It reads’’ Overall, 20% of adolescents in Africa met the current WHO physical activity recommendation. This finding, for example, is in line with the findings of earlier global investigations^2,15^. Physical inactivity among adolescents aged 11 to 17 years was shown to be significantly high in the global pooled study of 298 population-based surveys comprising 1.6 million adolescents^2^ . Xu et al and McMahon et al also reported similarly high rates of physical inactivity in adolescents in their respective multinational studies^16,17^. These estimates present a worrying prospect given the implications of sedentary behavior and physical inactivity on the development of coronary heart diseases and a host of other non-communicable diseases^18^’’.

**Comment 17:** 2nd paragraph, I believe this sentence you be in Introduction instead.

**Authors’ response.** The authors would like to thank the reviewer for the comment. The sentences now moved to the introduction now.

**Comment 18:** 3rd paragraph, do you have any hypothesis why gender gap in Benin, Algeria, Tunisia and Mozambique are more pronounced than other African countries in this study?

**Authors’ response:** Thanks for the comment. The authors did not hypothesized why gender is more pronounced in theses countries, the outcome was purely exploratory. However, potential reasons for this outcome have further been discussed. It reads ‘’ In our sample, widening gender gap were particularly pronounced in Benin, Algeria, Egypt, Libya Tunisia, and Mozambique. Generally, Ricardo and colleagues attributed this widening gap to stereotypes, body image insecurity, and cultural acceptability due to intrinsic sociocultural norms hence making girls less enjoy and confidence to engage in sport related activities^6^. Additionally, it was observed that most north African countries largely form the widening gender gap with respect to physical activity participation. This could be ascribed to various traditional and religious gender norms that persist in these countries such as the need for unmarried women to be accompanied in public places, conservative attires unsuitable for related physical activity engagement, and scarcity and accessibility of specific gender fitness facilities due to safety concerns^19^. Consequently, there are greater freedom for boys to engage more in physical activity than their girls counterpart^20^. Narrowing the gender gap in Africa could commence with offering more opportunities for safe, better access, investment and shifting socio cultural norms will increase the overall physical activity engagement and achieve the WHO’s global physical activity target by 2030^1,2,21^’’

**Comment 19:** 3rd paragraph, 6th sentence, this sentence does not quite support the whole paragraph whole argument since it is talking about behaviour modification. I would suggest focusing on what could drive the gender gap in these countries. Also, it would be helpful to show and discuss countries which are more equal or even have female adolescents engaging in more physical activity compared to the male counterparts.

**Authors’ response:** Thanks for the comment. The authors have taken the reviewer’s comment into consideration and have rectified and re-phrased the 6^th^ sentence on the 3^rd^ paragraph. Furthermore, potential reasons driving the gender gap in these countries have been discussed. Furthermore, authors have discussed countries which have narrowed/similar gender gap.

**Comment 20:** 3rd paragraph, last sentence, while this sentence is more supportive of the whole paragraph, but the conclusion on social, cultural, and so on is disconnected from the study.

**Authors’ response:** Thanks for the comment. The authors have taken the reviewer’s comment into consideration and accordingly rectified the disconnected sentence. It now reads ‘’ Addressing the rising burden of insufficient physical activity in adolescent girls and narrowing the gender gap in Africa could commence with offering more opportunities for safe, better access, investment and shifting socio cultural norms will ultimately increase the overall physical activity engagement and achieve the WHO’s global physical activity target by 2030^1,2,21^’’

**Comment 21:** There is no discussion on heterogeneity? Could the authors consider any other factors that may bias findings? For eg were all included schools in urban areas? And if not, how do these findings differ between urban and rural? And across different socio-economic levels? This is the most significant weakness of the paper and it makes it challenging to rigorously interpret the findings.

**Authors’ response:** The authors will like to thank the reviewer for this important comment. As recommended by the reviewer, further analyses have been performed. The authors have explored the possible source of heterogeneity through sub-group and meta-regression analyses and have discussed accordingly in the results and the discussion sections. The paragraph reads ‘’ Heterogeneity between the surveys’ countries in the pooled prevalence of sufficient physical activity level was high, extending I^2^ >87%. As a result, sources of heterogeneity were further explored through sub-group and meta-regression analyses with respect to HDI, countries’ economic health, sub-region, and survey years. The only possible source heterogeneity was the survey years. For instance, in the sub-group analysis, surveys performed between 2003-2010 had sufficient physical actively level of 18% as compared to survey conducted between 2011-2017 (i.e., 22%). Similarly, the multivariable meta-regression showed increased trends across the survey years’’. …... ……finally, due to the nature of the data; rural vs. urban could not be stratified and analyzed

Conclusion
**Comment 22:** Last sentence, same concern the recommendation is disconnected from the study.

**Authors’ response:** Thanks for the comment. The last sentence of the conclusion is now modified. The conclusion now reads ‘**’** The current study based on over 64,000 in-school adolescents, is the first to estimate physical activity level across 23 African countries. Only 20% of in-school adolescents met the WHO’s recommended physical activity level. Generally, adolescent girls in Africa are less active than adolescent boys. Addressing the rising burden of insufficient physical activity in adolescent and narrowing the gender gap could ultimately increase the overall physical activity engagement and achieve the WHO’s global physical activity target by 2030 among in-school adolescents in Africa.’’
